# Clinical Research into Central Nervous System Inflammatory Demyelinating Diseases Related to COVID-19 Vaccines

**DOI:** 10.3390/diseases12030060

**Published:** 2024-03-20

**Authors:** Mei-Yun Cheng, Hsuan-Chen Ho, Jung-Lung Hsu, Yi Wang, Linyi Chen, Siew-Na Lim, Ming-Feng Liao, Long-Sun Ro

**Affiliations:** 1 Section of Epilepsy, Department of Neurology, Chang Gung Memorial Hospital at Linkou Medical Center and Chang Gung University College of Medicine, Taoyuan 333, Taiwan; cheng-mei-yun@yahoo.com.tw (M.-Y.C.); siewna@adm.cgmh.org.tw (S.-N.L.); 2 Department of Medical Science, National Tsing Hua University, Hsinchu 300, Taiwan; lchen@life.nthu.edu.tw; 3 Department of Neurology, Chang Gung Memorial Hospital at Linkou Medical Center and Chang Gung University College of Medicine, Taoyuan 333, Taiwan; dolphin880131@gmail.com (H.-C.H.); tulu@adm.cgmh.org.tw (J.-L.H.); mingfengliao@hotmail.com (M.-F.L.); 4 Department of Neurology, New Taipei Municipal TuCheng Hospital, Chang Gung Memorial Hospital, Chang Gung University, New Taipei 236, Taiwan; 5 Graduate Institute of Mind, Brain and Consciousness, Taipei Medical University, Taipei 110, Taiwan; 6 Brain and Consciousness Research Center, Shuang Ho Hospital, New Taipei 235, Taiwan; 7 Institute of Molecular Medicine, National Tsing Hua University, Hsinchu 300, Taiwan; ton070735@gmail.com

**Keywords:** acute disseminated encephalomyelitis, central nervous system inflammatory demyelinating diseases, COVID-19 vaccines, myelin oligodendrocyte glycoprotein antibody-associated disease, neuromyelitis optica spectrum disorders, transverse myelitis

## Abstract

Various vaccines have been developed in response to the SARS-CoV-2 pandemic, and the safety of vaccines has become an important issue. COVID-19 vaccine-related central nervous system inflammatory demyelinating diseases (CNS IDDs) have been reported recently. We present one case of AstraZeneca vaccine-related myelin oligodendrocyte glycoprotein (MOG) antibody-associated disease and a literature review of another 78 patients published from January 2020 to October 2022. Patients were divided into three vaccine types (viral vector, mRNA, and inactivated vaccines) for further analyses. Among 79 patients with COVID-19 vaccine-related CNS IDDs, 49 (62%) cases received viral vector vaccines, 20 (25.3%) received mRNA vaccines, and 10 (12.7%) received inactivated vaccines. Twenty-seven cases (34.2%) were confirmed with autoantibodies, including fifteen patients (19%) with anti-MOG, eleven (13.9%) with anti-aquaporin 4 (AQP4), and one (1.3%) with both antibodies. Significantly, more males developed CNS IDDs post viral vector vaccines compared to mRNA and inactivated vaccines. Patients receiving mRNA vaccines were older than those receiving other types. Furthermore, mRNA and inactivated vaccines correlated more with anti-AQP4 antibodies, while viral vector vaccines showed higher MOG positivity. This research suggests potential associations between COVID-19 vaccine-related CNS IDDs and gender, age, and autoantibodies, contingent on vaccine types. Protein sequence analysis implies similarities between the S protein and AQP4/MOG. Further studies may elucidate the mechanisms of CNS IDDs, aiding vaccine selection for specific types.

## 1. Introduction

Diseases of myelin sheaths in the central nervous system (CNS) can be divided into two categories: genetic dysmyelinating diseases with abnormal myelin formation and acquired inflammatory demyelinating diseases, or so-called central nervous system inflammatory demyelinating diseases (CNS IDDs). CNS IDDs include multiple sclerosis (MS), neuromyelitis optica spectrum disorders (NMOSDs), myelin oligodendrocyte glycoprotein antibody-associated disease (MOGAD), acute disseminated encephalomyelitis (ADEM), optic neuritis (ON), and transverse myelitis (TM) [1].

NMOSDs and MOGAD are both related to autoantibodies, which target aquaporin 4 (AQP4) and myelin oligodendrocyte glycoprotein (MOG), respectively. The incidence and prevalence of NMOSDs is about 0.28–0.73 per 100,000 person-years and 0.52–10 per 100,000 people, respectively [2]. The variability in epidemiological data on NMOSDs may be attributed to differences in study designs, geographical regions, and ethnicities. NMOSDs are more common in East Asian, African, and Latin American populations compared to other Western populations, and the prevalence is 2.3 to 7.6 times higher in women than in men [3]. Previous studies in Europe estimated that the incidence of MOGAD is around 0.16–0.34 per 100,000 person-years, and the prevalence is 2 per 100,000 people [4,5]. The median age of onset is from the early- to mid-thirties with a slight predominance in females (57–68%) [6,7,8]. The clinical manifestations of MOGAD are diverse and can include one or a combination of the following diseases: ON, TM, and ADEM [9]. ON was the most common symptom (54–68.5%), followed by TM (27–30%) and ADEM or ADEM-like presentation (18–25%) [6,8,10]. The initial presentation of MOGAD in children and adults was also found to be different: ADEM was most common in children and ON was primarily found in adults [7].

ADEM is characterized by monophasic multifocal neurologic symptoms, and its diagnosis requires exclusion of MS, NMOSDs, MOGAD, or other demyelinating diseases [11]. ADEM is primarily regarded as a post-infectious disease, whereas vaccine-related ADEM is a rare condition. According to a prior systematic review, determining the incidence of CNS IDDs in confirmed COVID-19 infections posed challenges [12]. In the Taiwan Centers for Disease Control and Prevention (CDC) reporting system, it is also not possible to obtain the exact incidence of CNS IDD after COVID infection. Furthermore, there is a need for clarification regarding the severity and treatment response of CNS IDDs following COVID-19 infection compared to those without the virus [12]. It has been published that <5% of ADEM cases are related to vaccination for diseases such as rabies, measles, mumps, smallpox, or Japanese B encephalitis [13]. However, a recent study in the United States reported that there were no statistically significantly increased risks of ADEM after vaccination for 5–28 days, except for tetanus, reduced diphtheria, and acellular pertussis (Tdap) [14]. Another study provided additional evidence indicating no association between ADEM and different vaccine types, including Tdap [15]. The controversial relationship between vaccination and ADEM remains ambiguous, and large-scale epidemiologic data and clinical studies are required to confirm their association.

During the outbreak of the severe acute respiratory syndrome coronavirus 2 (SARS-CoV-2) pandemic, vaccines were one of the most effective ways to prevent infection. The mechanisms of COVID-19 vaccines include viral vectors (AstraZeneca, Janssen, Sputnik V, CanSino), mRNA (Pfizer-BioNTech and Moderna), inactivated vaccines (Sinovac, Sinopharm, Covaxin), and protein subunit vaccines (Medigen). Various side effects of COVID-19 vaccines have been widely reported recently. Although the distinct mechanism has not yet been elucidated, some adverse events may be related to specific types of vaccines. For example, vaccine-induced immune thrombotic thrombocytopenia (VITT) and Guillain–Barre syndrome are generally considered to be related to viral vector COVID-19 vaccines [16,17,18,19]. An elevated risk of myocarditis was observed in a population of young men who received mRNA COVID-19 vaccines [20]. CNS IDDs, such as TM, NMOSD, ADEM, or ON, have been reported after receiving AstraZeneca [21,22,23,24,25,26,27,28], Janssen [29], Sputnik V [30], Pfizer-BioNTech [31,32,33,34,35], Moderna [36,37,38,39,40], Sinovac [41,42], and Sinopharm [43,44] vaccines and an unknown inactivated vaccine [45].

We speculate that COVID-19 vaccines may induce CNS IDDs in a small subset of the population. Additionally, different types of COVID-19 vaccines may lead to different clinical manifestations, laboratory, or imaging characteristics of CNS IDDs. We now present a patient who developed MOGAD following COVID-19 vaccination and recruitment of relevant cases from the updated literature for comparison and analysis.

## 2. Materials and Methods

### 2.1. Case Report

Clinical features, brain and spinal MRIs, and laboratory data were obtained from the index case’s medical chart records.

### 2.2. Literature Review

To analyze the clinical presentations and MRI findings of patients with CNS IDDs after COVID-19 vaccination, a literature review was conducted using PubMed, EMBASE, Google Scholar, Ovid, and SCOPUS. Published articles and preprints of cases between January 2020 and October 2022 were included to obtain sufficient data, particularly brain and spinal MR imaging. The Medical Subject Heading (MeSH) terms “acute disseminated encephalomyelitis (ADEM)”, “myelin oligodendrocyte glycoprotein antibody-associated disease (MOGAD)”, “neuromyelitis optica spectrum disorders (NMOSDs)”, “optic neuritis”, and “myelitis” with “COVID-19 vaccines” were used to retrieve all accessible articles. We aimed to identify cases with first onset of CNS IDDs related to COVID-19 vaccination. Those diagnosed with relapses of MS, SARS-CoV-2 infection, Guillain–Barre syndrome, and CNS infection were excluded. We recorded the clinical features, past history, laboratory findings, brain/spinal MR imaging, treatment, and outcomes of our index patient in addition to previously reported cases of CNS IDDs after receiving COVID-19 vaccines. The lesions on brain MRIs were classified as cortical, deep gray matters, white matters (periventricular, subcortical, periaqueductal), brainstem, and with or without gadolinium enhancement. The lesions on spinal MRIs were divided into long-cord (≥3 segments) or short-cord (<3 segments), and with or without gadolinium enhancement.

In total, 79 cases (including our index case of MOGAD) diagnosed with CNS IDDs after COVID-19 vaccination were identified across 45 articles [21,22,23,24,25,26,27,28,29,30,31,32,33,34,35,36,37,38,39,40,41,42,43,44,45,46,47,48,49,50,51,52,53,54,55,56,57,58,59,60,61,62,63,64,65] (Table 1). Among them, the proportion of females (54.4%) was higher than that of males (45.6%), with average ages of 46.2 and 48.5 (*p* = 0.524), respectively. The majority of patients developed symptoms after the first dose (77.2%) or second dose (21.5%) of the COVID-19 vaccine, while one patient (1.3%) developed CNS IDDs after the third dose. The mean time of onset from vaccination to clinical symptoms was approximately 12.2 days (range 1–42 days). Twenty-one patients (26.6%) presented with ADEM or ADEM-like symptoms, nineteen cases (24.1%) with myelitis, fifteen (19%) with MOGAD, thirteen (16.5%) with NMOSDs, ten (12.7%) with ADEM with myelitis, and one (1.3%) with ON. A total of 27 patients (34.2%) had autoantibodies, including 15 patients (19%) with anti-MOG antibodies, 11 patients (13.9%) with anti-AQP4 antibodies, and 1 patient (1.3%) with both anti-MOG and anti-AQP4 antibodies. In the CSF examination of the 79 cases, the average WBC was 63.5 cells/µL with lymphocyte predominant, and the average total protein was 69.5 mg/dL. Additionally, oligoclonal bands were positive in 13 cases (16.5%).

### 2.3. Statistical Analysis

SPSS 26.0 (IBM Corp., Armonk, NY, USA) was used for statistical analyses. Categorical variables were compared using Pearson’s chi-square test or Fisher’s exact test. One-way analysis of variance (ANOVA) was used to compare unpaired groups. An independent samples *t*-test was applied for continuous variables. The level of significance was set at *p* < 0.05. All tests were two-tailed.

## 3. Results

### 3.1. Case Report

**Figure 1:**
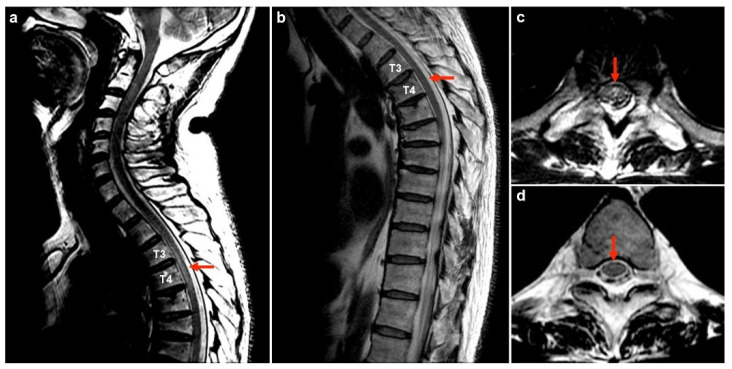
Spinal MRI: On the 6th day after onset, an intramedullary spotty lesion with T2 hyperintensity at T3 to T4 was shown (red arrows: (**a**) sagittal view. (**c**) axial view). Five months later, the lesion over T3 to T4 was totally resolved on T2 FLAIR images (red arrows: (**b**) sagittal view. (**d**) axial view).

**Figure 2:**
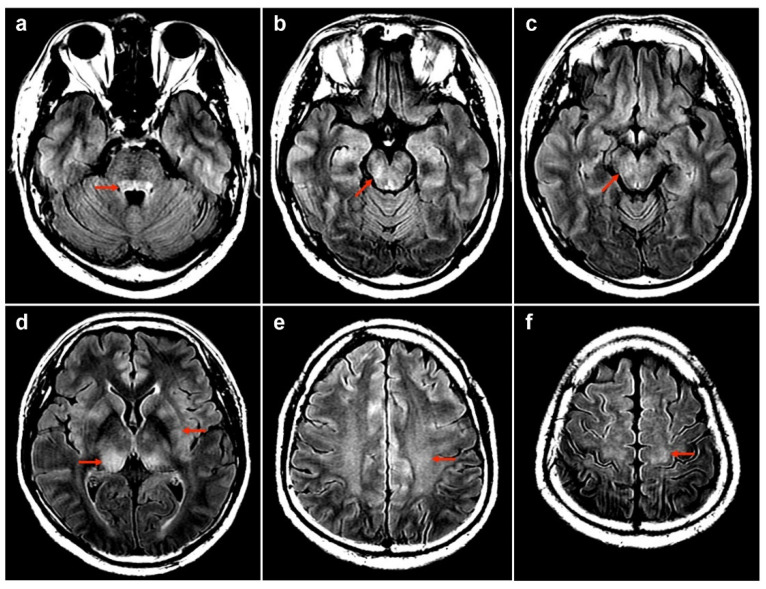
Brain MRI: Bilateral poorly demarcated T2 FLAIR hyperintensities at brainstem (**a**–**c**), pulvinar thalami (**d**), putamen (**d**), centrum semiovale (**e**), and subcortical white matters (**f**) are marked by red arrows in the image.

Our report describes a 50-year-old man who has chronic hepatitis B virus (HBV) disease and with no other comorbidities (Case No. 1 in Table 1). After receiving the first dose of the COVID-19 AZD1222 vaccine (AstraZeneca) via a 0.5 mL intramuscular injection into the deltoid muscle [66], the patient experienced a mild headache and general weakness that persisted for 2 days and then spontaneously subsided. On the 13th day following vaccination, he experienced an acute onset of a headache which was exacerbated upon waking up in the morning. In addition, fever, bilateral lower limb numbness, low back pain, confusion, slow response, unsteady gait, constipation, and urine retention requiring insertion of a Foley catheter developed over the following 4 days. Neurologic examination revealed Glasgow coma scale E4V5M6, but with episodic lethargy and obtundation. Impaired proprioception in the bilateral lower limbs leading to an ataxic gait and decreased pin-prick sensation below the T4 level were observed. Motor function and deep tendon reflexes were normal, and the Babinski reflex was a bilateral plantar flexion. Autonomic dysfunction led to constipation and urine retention. Cervical and thoracic spinal MRI revealed T2 hyperintensity in the spinal cord at the T3 to T4 levels without contrast enhancement, resembling focal myelitis (Figure 1a,c, 19th day post-vaccination). Brain MRI revealed symmetric, poorly demarcated hyperintensities involving the brainstem, bilateral pulvinar thalami, putamen, and centrum semiovale, with subcortical white matter fluid-attenuated inversion recovery (FLAIR) and T2-weighted imaging (T2WI) without gadolinium enhancement (Figure 2, 20th day post-vaccination). Blood tests revealed leukocytosis (11,000 cells/mL) with neutrophil predominance (85%) and elevated C-reactive protein (CRP) (48.49 mg/L). Urinalysis for intoxication was negative. Cerebral spinal fluid (CSF) analysis showed lymphocytic predominant pleocytosis (175 cells/µL, 99% lymphocytes), elevated total protein (78.1 mg/dL), and low CSF/serum glucose ratio (0.47).

**Figure 3:**
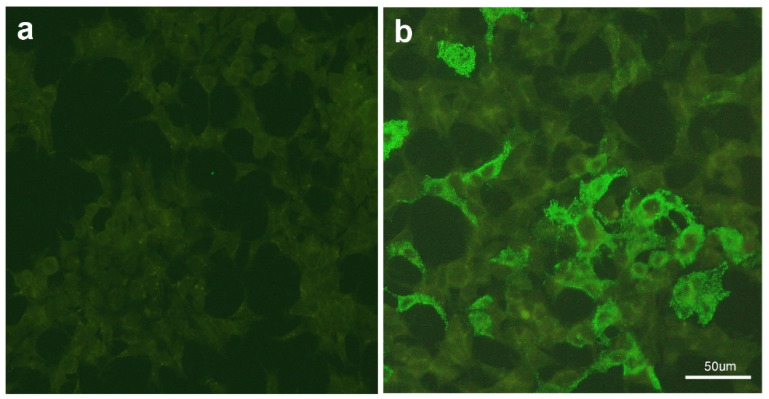
Cell-based immunofluorescence assay (Euroimmun, Lübeck, Germany): Indirect immunofluorescence test was used on HEK-293 cells transfected with plasmids containing MOG. Compared to the negative control (**a**), positive fluorescence was observed over the cell membrane and cytoplasm in transfected HEK-293 cells after applying the diluted (1:10) patient’s serum (**b**) and FITC-labeled secondary anti-human IgG antibody at 20× magnification.

He was administered empiric antibiotics (ceftriaxone and vancomycin) and antiviral (acyclovir) treatment initially, which were discontinued because results for bacterial culture and viral examination of CSF and blood were negative. HBV DNA was positive (439 IU/mL) and HBsAg was reactive (1484 IU/mL). Other laboratory results, including COVID-19 (nasopharyngeal swab for SARS-CoV-2-RT-PCR), Japanese encephalitis virus (JEV), herpes simplex virus (HSV), cytomegalovirus (CMV), Enterovirus, Epstein–Barr virus (EBV), varicella zoster virus (VZV), human immunodeficiency virus (HIV), cryptococcus, tuberculosis, syphilis, hepatitis C, and mycoplasma, were all negative. Further tests for vitamin B12 deficiency, vasculitis, connective tissue diseases, and tumor markers were unremarkable. Anti-AQP4 antibodies and oligoclonal bands were not found, but anti-MOG antibodies were detected in the serum by cell-based assays (CBA) using the commercially available kit (NMOSD Screen 1 EUROPattern, Euroimmun, Luebeck, Germany) (Figure 3). An indirect immunofluorescence test on HEK-293 cells transfected with plasmids containing myelin oligodendrocyte glycoprotein (MOG) was conducted. The initial 1:10 diluted serum was applied to transfected HEK-293 cells with plasmids containing MOG. Following a 30 min incubation, FITC-labeled secondary anti-human IgG antibody was added to the reaction field and incubated for an additional 30 min. Evaluation was performed using a fluorescence microscope. A positive antibody result was indicated by corresponding cytoplasmic and/or cell membrane smooth-to-fine-granular fluorescence in transfected HEK-293 cells (Figure 3b).

Pulse therapy with methylprednisolone (1 g per day for 5 consecutive days) was administered [67], followed by oral prednisolone (1 mg/kg/day) with gradual tapering. His consciousness and response became clear, and proprioception and pin-prick sensation gradually improved from the 2nd day after pulse therapy. The patient was discharged with total recovery of proprioception, pin-prick sensory level receding to T10 level, and improvement in constipation. The Foley catheter was successfully removed on the 38th day post-vaccination. Spinal MRI 5 months after vaccination revealed total resolution of T3 to T4 myelitis (Figure 1b,d). All neurological deficits have fully recovered. Anti-MOG antibodies in serum remained positive after 7 months of follow-up.

### 3.2. Literature Review

COVID-19 vaccines with different mechanisms of action may lead to different manifestations of CNS IDDs. These numbers and percentages reported here are derived solely from our index case and reviewed case reports, and do not represent the true incidence percentages in recipients of each type of vaccine. Among the 79 cases, we categorized the vaccines into three major vaccine types, including viral vector (49 cases, 62%), mRNA (20 cases, 25.3%), and inactivated vaccines (10 cases, 12.7%), to analyze their clinical presentations and laboratory examinations of CNS IDDs (Table 2). Compared to viral vector vaccines, CNS IDDs were more commonly observed in female patients receiving mRNA or inactivated vaccines (*p* = 0.027). The mean age of onset of patients receiving mRNA vaccines was much older than those receiving viral vector or inactivated vaccines (*p* = 0.002). The vast majority of patients in the viral vector vaccine type developed CNS IDDs after the first dose. Approximately two-thirds of the mRNA vaccine type experienced CNS IDDs after the first dose, and one-third after the second dose. In the inactivated vaccine type, there was one patient who developed CNS IDDs after the third dose. The time interval between vaccination and clinical presentations of CNS IDDs in patients receiving mRNA vaccines appears to be shorter than the other two types, although it is not statistically significant. There was no significant difference in the clinical presentations of CNS IDDs among these three types. Patients receiving mRNA or inactivated vaccines were more commonly found to have anti-AQP4 antibodies, while those receiving viral vector vaccines were frequently associated with anti-MOG antibodies (*p* = 0.044). On the other hand, a higher rate of CSF pleocytosis with lymphocyte predominant was observed in patients administered viral vector and mRNA vaccines (*p* = 0.004). Spinal cord lesions with gadolinium enhancement were most commonly found in patients who received mRNA vaccines (*p* = 0.015). There was no significant difference in the distribution of brain lesions or LETM (≥3 contiguous vertebral segments) between the three types. Among the 79 patients, 23 had documented past history, with hypertension and diabetes being the most common, each affecting patients (Table 1). The next most common diseases were hyperlipidemia and autoimmune diseases, each with four patients. Nevertheless, there was no statistically significant correlation between past history and the occurrence of CNS IDDs due to receiving different types of vaccines (data not presented in Table 2).

The majority of patients (86%) received first-line immunotherapy, including steroid pulse therapy, plasmapheresis/plasma exchange, or IVIG. Ten patients (13%) underwent additional second-line immunotherapy, such as cyclophosphamide or Rituximab. Among the 50 patients with available prognosis data, 3 individuals died (3.8%), while the remaining patients either fully recovered (20 patients) or showed improvement (27 patients).

### 3.3. Protein Sequence Analysis

**Figure 4:**
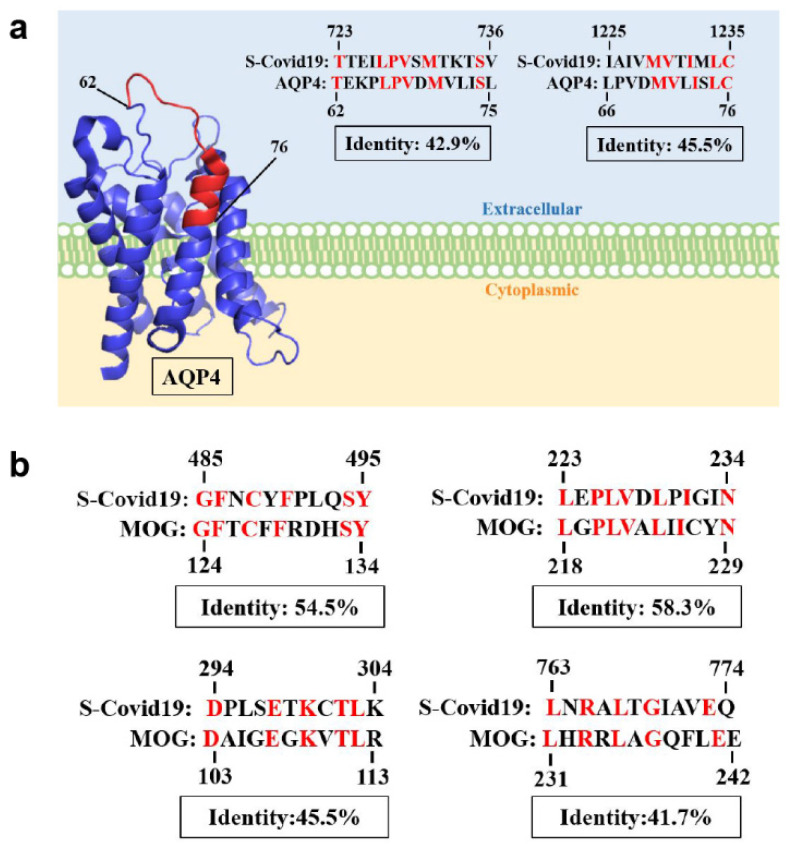
S-Covid 19 refers to the spike protein of severe acute respiratory syndrome coronavirus 2 (SARS-CoV-2), retrieved from the Protein Data Bank (PDB) under the accession number 6X29. Aquaporin 4 (AQP4) has a well-documented 3D structure with established structural domains, sourced from the PDB with the accession number 3GD8 (**a**). Notably, the extracellular loop of the AQP4 transmembrane protein can function as an antigen or be recognized by antibodies. The 3D structure of MOG is unknown (**b**). The Expasy SIM protein sequence alignment tool was utilized for analysis (https://web.expasy.org/sim/ accessed on 6 June 2023). Additionally, we employed PyMOL for protein structural analysis. The red-highlighted structure represents regions of high similarity between the two proteins.

By aligning proteins using protein sequence databases, it is possible that the S protein shares similar protein sequences with AQP4 and MOG (Figure 4).

## 4. Discussion

During the global SARS-CoV-2 pandemic, universal vaccination was a key factor in preventing infection and avoiding critical illness [68]. The overall safety and tolerability of COVID-19 vaccines has been shown to be generally acceptable in patients with underlying CNS IDDs, but there were still some patients (16.7%) who reported worsening of their symptoms after vaccination during the first week, with rapid resolution within 3 days [69]. The cases we reviewed in our study resulted from exposure to COVID-19 vaccines and they did not have a past history of CNS IDDs. The decision whether to receive the second or booster shots of COVID-19 vaccines, or to shift to another kind of vaccine, are important clinical issues.

According to statistics from the Centers for Disease Control in Taiwan [70], as of April 2023, the COVID-19 vaccine coverage rate was 94% for the first dose, 89% for the second dose, and 76.7% for additional doses. Most people received mRNA or viral vector vaccines, including Moderna (42.7%), Pfizer–BioNTech (29.2%), AstraZeneca (22.5%), Medigen (4.5%, a type of protein subunit vaccine produced in Taiwan), and Novavax (0.9%). Various adverse effects were reported. Six cases of ADEM (not included our index case), two cases of NMOSD, thirteen cases of ON, and four myelitis cases suspected to be related to COVID-19 vaccination were reported on the Taiwan CDC website, but not formally published [70]. Thrombosis with thrombocytopenia syndrome (TTS, also known as VITT) was suspected in 74 cases (86% of them received AstraZeneca, 8% received Moderna, 4% received Pfizer, and 1% received Medigen) [70]. In Taiwan, the first case of VITT after AstraZeneca vaccination was diagnosed in May 2021 at Chang Gung Memorial Hospital, a tertiary medical center. The patient presented with severe headaches, thrombocytopenia, and abdominal pain without neurological deficit. Brain CT revealed lacunar infarction in the right centrum semiovale. His platelet count normalized after treatment with high-dose IVIG (2 g/kg for two consecutive days) and he was discharged without further thrombotic or hemorrhagic events [71].

Our study provides a detailed case presentation of typical MOGAD clinical manifestations after receiving the AstraZeneca COVID-19 vaccine. A comprehensive comparison was conducted with previously published case reports. All 79 cases were excluded for concurrent COVID-19 infection. Since this complication of CNS IDDs following COVID-19 vaccination remains rare, the precise incidence rate has not yet been specified in pharmaceutical product documentation or registries, such as the Vaccine Adverse Event Report System (VAERS). The number of CNS IDDs related to COVID-19 vaccines may be underestimated. Factors contributing to this uncertainty include the widespread administration of COVID-19 vaccines, patients displaying relatively mild symptoms, lack of relevant diagnostic tools in resource-limited areas, the requirement for case reports to undergo peer review, and the unknown efforts made to contact the authors of published cases for additional information.

The pathophysiology of vaccine-induced CNS IDDs remains unclear. Many studies have suggested the molecular mimicry theory. For example, amino acid similarities between small HBV surface antigen (SHBsAg, the target of HBV non-infectious viral subunit vaccine) and MOG have been analyzed, displaying 66–100% homology in several sequences [72]. This finding suggests the possibility of immunological cross-reactivity. H1N1 and HPV vaccines have been identified with suspicious overlapping targets, which may result in certain autoimmune diseases [73]. While there is currently no evidence of molecular mimicry related to COVID-19 vaccines, the detection of anti-MOG antibodies and anti-AQP4 antibodies after receiving COVID-19 vaccines suggests potential similarities in the amino acid sequences of MOG, AQP4, and the spike (S) protein of SARS-CoV-2 virus or other unidentified immunological and inflammatory mechanisms. Articles discussing the similarity in protein sequences between the S protein and MOG or AQP4 have not been previously proposed. Although the S protein shares similar protein sequences with AQP4 and MOG (Figure 4), there are still some critical factors that may affect the protein structure, including hydropathy, global charge, and volume modifications. These factors have the potential to induce variations in the three-dimensional structures during protein folding, thereby altering the binding affinity with antibodies targeting the S protein [74]. Further clinical studies and experiments are needed to clarify the association and pathogenic mechanisms between COVID-19 vaccines and CNS IDDs. Once the definite pathological mechanisms have been elucidated, CNS IDDs induced by COVID-19 vaccines may be avoided by the development of safer vaccines. A larger database is required for further investigation into the relationship between demographics and vaccine types. The occurrence of CNS IDDs may be reduced by employing more precise indications for each kind of COVID-19 vaccines.

There are several limitations in our study, including a small number of cases, selection bias, difficulty in accessing complete original patient data, inability to confirm pre-onset levels of AQP4 and MOG antibodies, and lack of long-term follow-up for recurrence. It is challenging to confirm a causal relationship between COVID-19 vaccines and CNS IDDs. Factors such as past history, other autoimmune comorbidities, genetics, adaptive and innate immunity, and brain lesions may influence the autoimmune response after receiving the vaccines.

The rare complication of CNS IDDs after vaccination should not restrain the use of vaccines during the COVID-19 pandemic. For those who develop vaccine-related neuroimmunological adverse effects, further treatment strategies should be considered. Although the short-term outcomes were primarily ideal among the patients listed in our review following appropriate treatment, the possibility of long-term sequelae or recurrence of CNS IDDs still exists. To our knowledge, NMOSD and MOGAD patients have a higher risk of relapse if seropositivity persists, and prolonged immunotherapy should be considered to prevent relapse [9,75]. Therefore, patients initially positive for autoantibodies are recommended for regular follow-up MRI and antibody testing. Attention is also required for seronegative cases because of the possibility of seroconversion or the existence of other yet undefined autoantibodies.

## 5. Conclusions

We observed that there were more male patients with CNS IDDs in the viral vector vaccine type and they were often accompanied by anti-MOG antibodies. Patients receiving mRNA vaccines were older and more commonly positive for anti-AQP4 antibodies. The current consensus is that the rare occurrence of CNS IDDs is not a contraindication to vaccination. More extensive studies with larger cohorts are necessary to elucidate the pathological mechanisms of vaccine-related CNS IDDs and can enable physicians to select safer and more appropriate vaccines for each individual type to reduce the risk of adverse effects.

## Figures and Tables

**Table 1:** Demographic, clinical features, and laboratory data of 79 patients with CNS IDDs after COVID-19 vaccines.

No	Age/Sex/PH	Vaccine/ Interval (D)	Dx	Serum		CSF						MRI		Tx	Outcome	Ref.
				AQP4	MOG	WBC	Lym (%)	Neut (%)	TP	Glu Ratio	OCB	Brain	Spine (Myelitis)			
1	50M/chronic HBV	1st AZ ^a^/13	MOGAD	−	+	175	99	0	78.1	0.47	NA	Bil thalami, pu, subcortical WM, brainstem	T3–T4	PT	R	Index case
2	45M	1st AZ/12	ADEM + SSM	−	−	44	P	NA	N	NA	+	Pons, R MCP, R thalamus; C+	Cervical, thoracic, conus medullaris; C+	PT	R	[21]
3	63M/DM, HL, IHD, Af	1st AZ/12	ADEM	−	−	2	NA	NA	69	N	+	Bil WM, bil CC, L thalamus, IC, L midbrain, lower pons, R MCP	N	PT + PP	E(D20)	[22]
4	45M/atopic dermatitis	1st AZ/8	LETM	−	−	481	NA	67	140	0.43	−	N	C3–T2	PT	I	[23]
5	25F	1st AZ/12	LETM	−	−	NA	NA	NA	54.6	0.55	−	N	T3–T5, T7–T8, T11–L1; C+ in T7–T8	PT	I	[24]
6	58M/DM, pulmonary sarcoidosis	1st AZ/7	LETM	−	−	11	100	0	162	0.54	+	NA	C1–T10; C+ in T3–T4, T9–T10	PT + PE	I	[25]
7	41M/DM	1st AZ/14	LETM	−	NA	11	100	0	44.3	NA	NA	N	T1–T6; C+	PT	R	[26]
8	44F	1st AZ/4	SSM	−	−	↑	P	NA	76.7	NA	−	N	T7–T8, T10–T11; C+ in T7–T8	PT	R	[27]
9	36M	1st AZ/8	SSM	−	−	NA	NA	NA	54	NA	NA	N	C6–C7; C+	PT	R	[28]
10	44F	1st Janssen ^b^/10	LETM	−	NA	227	96	0	43	N	−	N	C2 to upper thoracic	PT + PE	R	[29]
11	34M	2nd Sputnik V ^c^/21	NMOSD	+	−	↑	P	NA	↑	NA	−	3rd, 4th PV, thalamus, CC, optic chiasma	N	PP	I	[30]
12	88F/DM, AD	2nd Pfizer ^d^/29	ADEM	NA	NA	NA	NA	NA	NA	NA	−	Bil MCPs	NA	PT	R	[31]
13	56F/Post-infectious rhombencephalitis	1st Pfizer/14	ADEM-like ^i^	−	−	N	NA	NA	N	N	−	L cerebellar peduncle and L centrum semiovale	NA	Oral steroid	R	[32]
14	64M	1st Pfizer/18	NMOSD	+	NA	N	NA	NA	N	N	−	CC, L frontal and parietal WM; C−	Cervical to the conus; C+	PT + PE + RTX	I	[33]
15	38M	1st Pfizer/2	SSM	NA	NA	NA	NA	NA	62.1	N	NA	NA	T11–T12; C+	NA	NA	[34]
16	69F/cervical ca, HL, hypothyroidism	1st Pfizer/2	LETM	−	−	N	NA	NA	N	N	+	N	C3–C4 to T2–T3	PT	I	[35]
17	19F/atopic dermatitis, depression	1st Moderna ^e^/14	ADEM + LETM	−	−	294	91	1	64.8	NA	−	Bil hemispheres, pons, medulla, cerebellum; C+	Medulla to T11; C+	PT + PE	R	[36]
18	46F/vit B12 deficiency	1st Moderna/2	NMOSD	−	NA	NA	NA	NA	NA	NA	NA	N	C6–T2	PT	R	[37]
19	76F/HTN, vit B12 deficiency	1st Moderna/2	LETM	−	NA	15	NA	73	57.2	NA	−	N	C2–C5; C+ in C3	PT	I	[38]
20	67F/CAD, CKD, neuropathy	1st Moderna/1	LETM	−	−	2	NA	NA	56	0.61	+	Nonspecific WM	C1–C3; C+	PT + PP	I	[39]
21	63M	2nd Moderna/1	SSM	−	−	3	NA	NA	37	N	NA	Nonspecific bil corona radiata	Conus medullaris; C+	IVIG + PT	I	[40]
22	46F/Hashimoto’s thyroiditis	2nd Sinovac ^f^/30	ADEM-like	−	−	0	0	0	45	NA	−	L thalamus, bil corona radiata, L diencephalon, R parietal cortex	NA	PT	R	[41]
23	78F/DM, HTN, breast ca	2nd Sinovac/21	LETM	−	−	2	NA	NA	56	0.69	−	N	C1–T3	PT	I	[42]
24	24F	1st Sinopharm ^g^/14	ADEM	−	−	51	NA	NA	NA	NA	−	Bil temporal	NA	IVIG	I	[43]
25	71M/DM, HTN, IHD	1st Sinopharm/5	LETM	−	−	0	0	0	N	N	−	N	Cervico–medullary junction to C3	PT	I	[44]
26	50F	1st inactivated/3	NMOSD	+	−	31	NA	NA	N	N	−	Area postrema, bil hypothalamus	N	PT	I	[45]
27	65M	1st AZ/8	LETM	−	−	N	NA	NA	70	NA	−	NA	C4–C6	PT	R	[46]
28	68F/HTN, pancreatic ca	2nd Moderna/14	MOGAD	−	+	0	0	0	32	NA	+	R lateral pons, trigeminal nerve, MCP	NA	PT	I	[47]
29	59M	1st AZ/14	MOGAD	−	+	110	NA	NA	625	N	+	N	T7–L1	PT + PE	I	[48]
30	45M/allergic asthma	1st AZ/7	MOGAD	−	+	43	NA	NA	40.6	N	−	Bil subcortical, gray–white matter	T10–conus	PT	I	[49]
31	26M	1st AZ/20	MOGAD	−	+	184	NA	NA	88	NA	−	Bil MCPs, pons	C3–C6	PT	I	[50]
32	56F/HTN	1st AZ/2	ADEM-like	NA	NA	NA	NA	NA	NA	NA	NA	L parietal WM, CC	NA	Oral steroid	I	[51]
33	81M	1st Moderna/13	ADEM	NA	−	69	83%	NA	52	N	NA	R dorsal medulla, L pons, midbrain, thalami	NA	PT + IVIG + PP	E(D26)	[52]
34	63F/HL, hypothyroidism	1st Pfizer/7	NMOSD	+	−	33	91%	NA	57	NA	−	L thalamus	T6–T12	PT + PP	R	
35	54F/ITP	2nd Moderna/3	NMOSD	+	−	26	86%	NA	71	NA	−	N	T2–T9	PT	I	
36	55M	1st mRNA/21	ADEM	NA	NA	200	95%	NA	75	N	NA	Bil WM	NA	PT	R	[53]
37	36F	1st AZ/14	ADEM	−	−	59	NA	NA	40	N	+	Subcortical WM, PIC, pons, L MCP	N	PT	R	[54]
38	37M	1st Sinopharm/30	ADEM	NA	NA	2	NA	NA	56	0.61	−	L cerebral peduncle, bil pons, medulla	N	PT	R	[55]
39	27F/Rectovaginal fistula	2nd Pfizer/4	LETM	−	−	7	NA	NA	43	N	−	N	C5–C7	PT	I	[56]
40	61F/HTN, anxiety	1st Pfizer/5	ADEM	NA	−	N	NA	NA	61	N	−	Deep WM, brainstem, cerebellum	N	PT + IVIG	I	[57]
41	64M	1st AZ/10	ADEM	−	−	25	P	NA	NA	N	NA	Bil mesial temporal, hippocampus, MCPs	NA	PT + PP + RTX	R	[58]
42	64M	2nd AZ/20	ADEM + SSM	−	−	N	NA	NA	N	N	NA	Bil perirolandic cortex, corona radiata	T8–T9 dorsal	PT + IVIG + RTX	I	
43	46M	1st AZ/5	ADEM + LETM	−	−	63	NA	NA	52	N	NA	Bil MCP, pons, R paramedian medulla, L thalamocapsular	LETM	PT + PE	I	
44	42F	1st AZ/5	ADEM-like	−	−	N	NA	NA	N	N	NA	R temporal	NA	Oral steroid	R	
45	56F	1st AZ/10	ADEM-like	NA	NA	1	16%	20%	↑	N	NA	Subcortical WM, basal ganglia	NA	PT	R	[59]
46	44F/HL, hypothyroidism, renal stone, anxiety	1st mRNA/6	ADEM + LETM	−	NA	105	NA	NA	98	N	−	Multifocal PV lesions; C+ in L frontal WM	C3–C4 to thoracic with sparing C5–C6; C+ in T7–T8	PT + PP	I	[60]
47	70F	3rd Sinovac/ 7	NMOSD	+	NA	N	N	N	N	N	−	NA	C1–C7 and T1–T3	PT + PE + CP	E(M2)	[61]
48	26F	1st Sinovac/10	NMOSD	+	NA	N	N	N	N	N	−	N	C4–C5	PT + PE + RTX	I	[62]
49	46F	1st AZ/10	NMOSD	+	NA	N	N	N	N	N	−	R lateral medulla, PV	C2–C3	PT +AZT	I	
50	80M	2nd Pfizer/2	NMOSD	+	+	39	93%	NA	N	N	−	N	T3–T10	PT + PE + MMF	I	[63]
51	43F	2nd Pfizer/1	NMOSD	+	−	6	NA	NA	40.1	N	+	R ON, R periatrium, L crus cerebri	C1 to mid-thoracic	PT + PE + RTX	R	[64]
52	29F	1st AZ/11	MOGAD	NA	+	0	NA	NA	18	N	−	Long intraorbital segment of R ON	NA	PT + PP	NA	[65]
53	26F	1st Covaxin ^h^/11	LETM	−	−	207	NA	P	95.8	N	NA	NA	C2–L1	PT + PP	NA	
54	54F	1st AZ/14	ADEM-like	−	−	8	P	NA	77	N	NA	CC, PV, subcortical WM, infratentorial	NA	PT + PP	NA	
55	44M	1st AZ/7	MOGAD	NA	+	130	P	NA	38	N	NA	NA	Cervical and dorsal cord, conus	PT + PP	NA	
56	50F	1st AZ/28	SSM	−	−	2	P	NA	28	N	NA	NA	C6	PT	NA	
57	39M	1st AZ/14	MOGAD	NA	+	NA	NA	NA	NA	NA	NA	Long intraorbital segment of R ON	NA	PT	NA	
58	54M	1st AZ/14	MOGAD	NA	+	NA	NA	NA	NA	NA	NA	R pons	N	PT	NA	
59	34M	1st AZ/1	ON	−	−	2	P	NA	26	N	NA	R ON	NA	PT	NA	
60	35M	1st AZ/9	MOGAD	NA	+	58	P	NA	47.4	N	NA	Midbrain, pons, L MCP, PICs, thalamus, bil centrum semiovale	Cervical to conus	PT	NA	
61	20F	1st AZ/3	ADEM-like	−	−	NA	NA	NA	NA	NA	NA	Pericallosal, callososeptal, PV, fronto-parietal	NA	PT	NA	
62	31M	1st AZ/14	LETM	−	−	370	NA	P	174	N	NA	NA	Cervico–dorsal long segment	PT + PP + RTX	NA	
63	20F	1st Covaxin/1	ADEM + SSM	−	−	8	P	NA	24.9	N	−	Juxtacortical	C5	PT + PP	NA	
64	45F	1st AZ/21	MOGAD	NA	+	2	P	NA	52.3	N	+	Bil ON	N	PT + PP	NA	
65	33F	1st AZ/14	MOGAD	NA	+	105	P	NA	28.12	N	NA	Bil fronto-parietal	NA	PT	NA	
66	53F	2nd AZ/1	ADEM + LETM	−	−	6	P	NA	54.2	N	NA	Bil subcortical, PV, insular, cerebellum, brainstem	C5–C7 and T6–T7	PT	NA	
67	38M	2nd AZ/6	ADEM-like	−	−	6	NA	NA	67.8	N	NA	L MCP, R corona radiata	NA	PT	NA	
68	30M	1st AZ/14	ADEM + ON	−	−	4	50	NA	26.8	N	+	Bil subcortical, bil ON	NA	PT + PP + RTX	NA	
69	30F	1st AZ/15	ADEM + SSM	−	−	4	NA	NA	36	N	+	CC	C3	PT + PP + MMF	NA	
70	36M	2nd AZ/32	MOGAD	NA	+	720	80	NA	144.4	N	NA	Bil trigeminal n, pons	Obex to conus	PT + PP	NA	
71	27F	1st AZ/8	ADEM-like	−	−	Clear	NA	NA	27.7	N	NA	Bil PV	N	PT	NA	
72	60M	2nd AZ/14	ADEM	−	−	9	90	NA	68.3	N	−	R pons, midbrain, temporal, parietal, CC	NA	PT + MMF	NA	
73	23F	2nd AZ/7	ADEM + LETM	−	−	NA	NA	NA	NA	NA	−	R frontal horn and bil lateral ventricles	C2–C5 and T4 myelitis	PT	NA	
74	40M	1st AZ/10	MOGAD	NA	+	8	100	0	32	N	+	Pons, bil thalami, and R frontal cortex	C4–T3	PT + MMF	NA	
75	45M	1st AZ/10	MOGAD	−	+	44	44	NA	90.9	N	NA	Brainstem, supratentorial	Cervicodorsal cord	PT + PP	NA	
76	34F	2nd AZ/36	NMOSD	+	−	1	NA	NA	15.3	N	−	Dorsal aspect of medulla	NA	PT + PP + RTX	NA	
77	31M	1st AZ/42	ADEM + LETM	−	−	32	100	0	49.2	N	NA	Cervico-medullary junction, R frontal subcortical	C2–C5	PT + PP + MMF	NA	
78	52F	1st AZ/35	ADEM-like	−	−	2	NA	NA	40.5	N	NA	L frontal, insular, midbrain	NA	PT + PP + RTX	NA	
79	65F	1st AZ/42	NMOSD	+	−	17	NA	NA	49	N	NA	Frontal subcortical WM	T2–T11	PT + PP + MMF	NA	

Abbreviations: ↑: means elevated without specifying exact values. Af: atrial fibrillation. AD: Alzheimer’s disease. AZT: azathioprine. C+: with contrast enhancement. CC: corpus callosum. CKD: chronic kidney disease. CP: cyclophosphamide. DM: diabetes mellitus. E: expired. HBV: hepatitis B virus. HL: hyperlipidemia. HTN: hypertension. I: improvement. IC: internal capsule. IHD: ischemic heart disease. ITP: immune thrombocytopenic purpura. MCP: middle cerebellar peduncle. MMF: *Mycophenolate* mofetil. N: normal. NA: not available. ON: optic neuritis. P: predominant. PIC: posterior limb of internal capsule. PP: plasmapheresis. PT: steroid pulse therapy. PU: putamen. PV: periventricular. R: full recovery. RTX: rituximab. WM: white matter. ^a^ Oxford–AstraZeneca, marketed as Covishield. A kind of viral vector (chimpanzee adenovirus) vaccine. ^b^ Johnson & Johnson’s Janssen, a kind of viral vector (human adenovirus) vaccine. ^c^ A kind of viral vector (human adenovirus) vaccine. ^d^ Pfizer–BioNTech, marketed as Comirnaty. A kind of messenger RNA vaccine. ^e^ A kind of messenger RNA vaccine. ^f^ Marketed as CoronaVac. A kind of inactivated vaccine. ^g^ Also known as BBIBP-CorV. A kind of inactivated vaccine. ^h^ Also known as BBV 152. A kind of inactivated vaccine. ^i^ Clinical and MRI features are compatible with ADEM, but without presentation of encephalopathy.

**Table 2:** Differences between three major vaccine types.

	*Vaccine Types*			
	Viral Vector (n = 49)	mRNA (n = 20)	Inactivated (n = 10)	*p* Value
Sex, n (%)				0.027 *
Male	28 (57)	6 (30)	2 (20)	
Female	21 (43)	14 (70)	8 (80)	
Mean age of onset (S.D.)	44.3 (12.2)	58.1 (17.9)	44.8 (21.8)	0.002 ^#^*
Doses (1st/2nd/3rd)	41/8/0	13/7/0	7/2/1	0.042 *
Post-vaccination onset time (days)	13.6	8.1	13.6	0.086
Clinical presentations, n (%)				0.203
ADEM	13 (27)	5 (25)	3 (30)	
Pure myelitis	10 (20)	6 (30)	3 (30)	
MOGAD	14 (29)	1 (5)	0 (0)	
NMOSD	4 (8)	6 (30)	3 (30)	
ADEM with myelitis	7 (14)	2 (10)	1 (10)	
ON	1 (2)	0 (0)	0 (0)	
Serum Autoantibodies , n				0.044 *
Negative	31	14	7	
MOG	14	1	0	
AQP4	4	4	3	
MOG + AQP4	0	1	0	
CSF, n				
WBC count	74.2	57.1	30.1	0.605
Lym predominant (yes/no)	23/3	6/2	1/4	0.004 *
Elevated total protein (yes/no) ^@^	24/19	10/8	4/5	0.817
CSF/serum glu ratio (<0.6/>0.6)	4/33	0/13	0/8	0.295
Oligoclonal bands (+/−)	9/13	4/11	0/9	0.071
Brain MRI lesions, n				
Cortex (+/−)	7/33	1/16	2/5	0.329
Deep grey matters (+/−)	8/32	2/15	2/5	0.598
Subcortical white matters (+/−)	18/22	5/12	2/5	0.454
Periventricular white matters (+/−)	16/24	6/11	1/6	0.424
Periaqueductal white matters (+/−)	3/37	0/17	0/7	0.389
Brainstem (+/−)	20/20	7/10	2/5	0.532
Gadolinium enhanced (+/−)	1/9	2/9	0/5	0.562
Spine MRI, n				
Segments of cord lesions (≥3/<3)	20/8	12/2	4/2	0.143
Gadolinium enhanced (+/−)	6/6	7/1	0/4	0.015 *
Treatment, n				0.754
First-line immunotherapy	43	17	8	
First- and second-line immunotherapy	6	2	2	
Outcome, n				0.762
Recovery	11	7	2	
Improved	11	11	5	
Expired	1	1	1	

* Pearson chi-squared test, *p* < 0.05; ^#^ Independent *t*-test, *p* < 0.05; ^@^ Elevated total protein defined as total protein > 45 mg/dL.

## Data Availability

The datasets generated during and/or analyzed during the current study are available from the corresponding author.
